# Impact of COVID-19 on the utilisation of maternal and child health services in Peru at national and subnational levels: An interrupted time series analysis

**DOI:** 10.7189/jogh.14.05039

**Published:** 2024-12-20

**Authors:** Luis Huicho, Carlos A Huayanay-Espinoza, Rodrigo Valladares, Alvaro G Oviedo-Rios, Soleda S Ruiz-Lopez, Nadia Akseer, Abdoulaye Maïga, Alicia Matijasevich, Agbessi Amouzou

**Affiliations:** 1 Centro de Investigación en Salud Materna e Infantil, Universidad Peruana Cayetano Heredia, Lima, Peru; 2 Centro de Investigación para el Desarrollo Integral y Sostenible, Universidad Peruana Cayetano Heredia, Lima, Peru; 3 Facultad de Medicina, Universidad Peruana Cayetano Heredia, Lima, Peru; 4 Department of International Health, Johns Hopkins Bloomberg School of Public Health, Baltimore, USA; 5 Departamento de Medicina Preventiva, Faculdade de Medicina FMUSP, Universidade de São Paulo, São Paulo, Brazil

## Abstract

**Background:**

The resilience of Peru´s health system was weakened by a political crisis that started in 2016 and was further challenged by the coronavirus 2019 (COVID-19) pandemic. We assessed the indirect impact of the pandemic on the utilisation of essential maternal and child health (MCH) services in Peru at national and subnational levels.

**Methods:**

We assessed the trends in MCH services utilisation and the percentage change from 2018 to 2021, using routine health facility data. We used an interrupted time series analysis to quantify the impact of COVID-19 on the utilisation of health services.

**Results:**

The utilisation of most maternal and child health services dropped dramatically in 2020 after the outbreak. However, we observed a quick recovery in 2021, with service utilisation fairly similar or higher to the pre-pandemic period (2018–2019). The decrease was higher in the utilisation of antenatal care visit one or more (incidence rate ratio (IRR) = 0.79; 95% CI = 0.74–0.83) and antenatal care visits four or more (IRR= 0.76; 95% = 0.74–0.79) in 2020. The IRR showed a drop of 5, 6, 9, and 13% in the utilisation of skilled birth attendances, institutional deliveries, caesarean sections and postnatal care visits within two days of childbirth, respectively in 2020 in comparison to pre-pandemic service utilisation. In 2020 the utilisation decreased in all three natural regions, with the Rainforest being the most affected. In 2021 there was a recovery in all natural regions.

**Conclusions:**

The pandemic decreased the utilisation of essential maternal and child health services in Peru. This highlights the need to preserve the resilience of a health system both at central and local levels, to face more successfully future pandemics.

The coronavirus 2019 (COVID-19) pandemic forced governments all over the world to impose diverse restrictions in population movements and lockdowns in the attempt to limit the circulation of the virus. An array of public services was substantially limited, among them health services including essential health services for women, infants and children, which threatened to halt or even revert the progress achieved in reproductive, maternal, neonatal and child health at global and country level [1].

Different publications in several regions of the world have shown the impact of COVID-19 on the utilisation of maternal and child services. The evidence suggests that the pandemic affected the continuity of those services through the restriction in the delivery of diverse interventions along the continuum of care of mothers and children, decreased funding, diversion to COVID-19 related services, or decreased demand [1–6].

Under-five mortality rate, neonatal mortality rate and under-five stunting prevalence were reduced substantially within the last two decades in Peru, thanks to the equitable implementation of several multisectoral interventions, although great inequities remain, particularly between urban and rural areas, and between rich and poor segments of the population [7–10].

Despite the above-mentioned impressive progress achieved by Peru in terms of maternal and child health, its health system is fragmented and highly inequitable. It is a four-tier system that includes:

a) the public sector (Ministry of Health and department-level health facilities, and police and armed forces health facilities)

b) the social insurance system (EsSalud)

c) the private for-profit sector

d) the private not-for-profit sector (nongovernmental and religious organisations health facilities) [11].

The Ministry of Health is based on a subsidised scheme that covers the uninsured population, while EsSalud is financed through employers and employees contributions and covers the population with a formal employment. The public sector faces the chronic shortage of adequate infrastructure and equipment, and of a qualified and motivated workforce, constraints that are more dramatic in rural and remote areas of the country. There is wide consensus that public primary heath care facilities in Peru provide low quality of health care, with inadequate provision of drugs and supplies, which is explained in part by a very low proportion of the gross national income allocated to health [11].

The first COVID-19 cases in Peru were reported on early March 2020. The government declared the state of health emergency on 11 March 2020, imposing severe restrictions to the movement of people and to the delivery of public services. Then a progressive easing of restrictions was implemented since early May 2020. The COVID-19 vaccination process started in February 2021 and was expanded progressively. A second wave of COVID-19 was declared on 12 January 2021 that lasted until June 2021, but the restrictions imposed were much less severe.

A systematic assessment of the effects of COVID-19 on maternal, neonatal and child health services within the above described context of Peru is critical to understand the size of the disruption, lessons learned, and to generate evidence in support of measures to address similar pandemics in the future. Based on previous studies in other settings [2–6], we hypothesised that the COVID-19 pandemic had an indirect, immediate and temporary negative effect on the level and slope of utilisation of essential maternal and child health services in Peru, acting on a fragmented and inequitable health system. The objective of this study was to assess the indirect impact of the COVID-19 pandemic on the utilisation of essential maternal and child health services in Peru at national and subnational level using routine health facility data. We specifically aimed at assessing the COVID-19 effects on interventions encompassing the continuum of care from antenatal to postnatal periods.

## METHODS

### Setting

Peru is classified as an upper-middle income country with a population estimated in 33.726 million inhabitants in 2022 [12]. It is characterised by a democratic government system composed by the executive, the parliament and the judiciary system, with separation of power. The constitution regulating the social contract of Peru has seen several changes along the last few years, modifying the level of autonomy of those different government institutions, and resulting in a significant enhancement of the parliament´s attributions.This in turn has led to frequent impeachment processes of presidents of the republic and to a higher turnover rate of the executive cabinets, which have ultimately affected the stability of all the public sector bureaucracy and its capacity to provide essential services.

Peru has three natural regions, namely the Coast close to the sea, the mountainous Highlands and the Rainforest. The Coast is better-off and more urbanised, while the Rainforest and the Highlands, particularly their rural areas, concentrate the poorest segments of the country and most of the indigenous communities. The country is politically divided in 25 departments or regions with differing socioeconomic, geographical and cultural characteristics. Lima is the capital city and its population is currently above 11 million inhabitants [12]. Therefore we aimed at disaggregating our study analyses at the natural region level and at the departmental level, to provide a more detailed picture that goes beyond the national profile.

### Study design

We conducted a quantitative assessment by describing national and subnational trends of essential maternal and child health interventions and implementing an interrupted time series analysis to assess the indirect impact of COVID-19 on the utilisation of health services. The study period was January 2018 to December 2021, including a pre-COVID-19 period (2018–2019) and a COVID-19 period (2020–2021).

We performed a desk review and virtual interviews with people familiar with the Peruvian routine health information system, to obtain from them details of the data gathering and consolidation process, to get their viewpoint on the data reliability, and to learn in depth about the political context and the COVID-19 containing measures implemented by the government. We interviewed six persons, from the Ministry of Health, the Ministry of Development and Social Inclusion and fom multilateral organisations, who were active or former human resources on health and political aspects with emphasis on maternal and child health.

### Data sources and outcomes

We used routine health facility data extracted from the Peru health information system managed by the Ministry of Health. They are monthly data disaggregated by department over the period of 2018 to 2021. We used each department (political and administrative division of the country) as the primary unit of our analysis. The departments are grouped into three natural regions (Coast, Highlands and Rainforest). The focused health service indicators reflect the continuum of maternal and child health care, and include the following: number of antenatal care visit one or more, number of antenatal care visit four or more, number of institutional deliveries, number of births attended by a skilled attendant, number of ceasarean sections, and number of postnatal care visits within two days of childbirth.

### Quality data assessment

We assessed the reporting completeness, defined as the proportion of facilities that reported data for a given month out of the total number of facilities expected to report data. The facility reporting completeness index was assessed at district level and was computed as the unweighted average of monthly facility reporting rates. for antenatal care, institutional delivery, immunisation, outpatient consultations, and inpatient admissions. The adjustment for incomplete reporting was based on both the completeness of reporting and the level of service provision expected from non-reporting facilities using an adjustment factor. The adjustment factor ranges from 0 to 1. We used 0.25 as the adjustment value for the case of Peru.

Outlier detection was also part of the data quality assurance process. An outlier value was defined as any monthly data with a score higher or lower than 5 standard deviations from the median absolute deviation. The score was a modified Z-score which is a standardised score of observations measuring the deviation from the median, obtained by dividing the difference from the median by the median absolute deviation (MAD) calculated from the preceding three years. After that, we computed lower (LB) and upper (UB) bounds within which values are expected. Any data outcomes out of the bounds were considered as outliers. Outliers were corrected by calculating the median value of the calendar year. We calculated the LB and UB as follows:

LB = Median − 1.4826 × 5 × MAD

UB = Median + 1.4826 × 5 × MAD

MAD is computed as follows:

MAD = median (|Xi – X~|), where Xi is the value of the observation for a particular time period (year) and X~ is the median of the three preceding years.

We considered 1.4816 as a constant default value, based on the assumption of normal distribution of the data. That means that for a normal distribution, one standard deviation from the mean is about 1.4826 MADs.

The internal consistency metric was calculated as the ratio of one antenatal care visit/one dose of pentavalent vaccine, and the ratio of one pentavalent vaccine/three doses of pentavalent vaccine. A value within the reference range of 1.0–1.5 was considered an acceptable ratio.

### Statistical analysis

As part of the descriptive analysis, we determined the annual, quarterly and monthly trends of the number of health services provided during the period 2018–2021 at national level, by natural regions and at departmental level. We further determined the percentage of change in the number of health services provided in 2020 and 2021, with the average numbers observed during the period 2018 to 2019 as the baseline.

To quantify the impact of COVID-19 on the utilisation of essential maternal and child health services, we used an interrupted-time series approach [13,14]. We used the declaration of health emergency along with the implementation of COVID-19 control restrictions at the national level as the interruption event, which occurred in Peru on 11 March 2020. We specifically followed a before-after design without a control series, as the whole population was exposed to the COVID-19 pandemic and therefore a comparison unexposed study population group was not available. We anticipated an indirect, immediate and temporary negative impact of the pandemic on both the level and slope of the study outcomes. We accounted for autocorrelation and seasonality as potential time-varying confounders, as specified below.

We used the technique of generalised iterative weighted linear regression, incorporating a Poisson model, adequate for count data [15]. The reference period for this analysis is the counterfactual non-pandemic scenario, based on pre-COVID-19 trends, that is, the period from January 2018 to February 2020.

We run segmented regressions by month and for the overall 2020 and 2021 periods. These regressions require a variable that counts the number of periods since the start of the study period, as well as a variable that is a vector of dummies for each month and dummy variables for the months during the pandemic. The estimated coefficients represent the differences between what is reported and what is predicted by the model for the counterfactual scenario without pandemic, based on pre-COVID-19 trends [3].

The following equation specifies the segmented regression model for the overall 2020 and 2021 periods:

*Yt* = *β*_0_ + *β*_1_*T* + _β2_ _Xt_ + *β*_3_*TXt* + *β*_4_ *X_t_*_+1_ + *β*_5_*TX_t_*_+1_ + e

Where:

*Yt* represents the dependent variable or primary outcome (the number of involved maternal and health service) at a given time.

*T* represents the time since the start of the study. The unit represents the frequency of the data report (monthly in our case).

*Xt* is a dummy variable that takes a value of zero for the pre-intervention (pre-event) period and one for the post-intervention period (2020).

TXt is a continuous variable counting the number of months after the event, in 2020.

*Xt+1* is a dummy variable that takes a value of zero for the pre-intervention (pre-event) period and one for the post-intervention period (2021).

*TXt+1* is a continuous variable counting the number of months after the event, in 2021.

*β_0_* estimates the baseline level at *T = 0.*

*β_1_* estimates the change in outcome associated with a time unit increase (representing the underlying pre-intervention trend).

*β_2_* estimates the level change following the event, in 2020.

*β_3_* estimates the slope change following the event, in 2020.

*β_4_* estimates the level change following the event, in 2021.

*β_5_* estimates the slope change following the event, in 2021.

*et* is the standard error at a given time.

The following equation specifies the segmented regression model by month:

*Yt* = *β*_0_ + *β*_1_*T* + *β*_2_*Month* + *β*_3_*March2020* + ⋯ + *β*_n_*December2021* + *et*

Where:

*Yt* represents the number of services reported.

*T* is a variable that counts the number of periods since the beginning of the study period.

*Month* is a vector of dummies for each calendar month to account for seasonality.

*March2020–December2021* are dummy variables for the months during the pandemic.

We considered as statistically significant a *P*-value <0.05.

To adjust for autocorrelation, we used the heteroskedasticity and autocorrelation consistent errors method, which is a correction approach used in regression analysis to handle problems of heteroscedasticity and autocorrelation. Through the estimation of weighting matrices, it adjusts standard errors and statistical tests so that they are robust to autocorrelation [16].

To adjust for seasonality, we used the Fourier terms transformation, which allows capturing cyclical and repetitive patterns that may arise due to seasonality in the data. The Fourier terms helped us modeling regular fluctuations that may occur at specific monthly time intervals [17].

This study used existing routine aggregated health information on health services that is codified to guarantee anonymity, and therefore identification of individuals was not possible. The data are freely available upon request. Furthermore, interviews were conducted as part of the administrative duties of the interviewees, and viewpoints individually expressed were synthesised and did not include personal information that would compromise participants confidentiality. Consequently, written informed consent was not required.

## RESULTS

**Figure 1:**
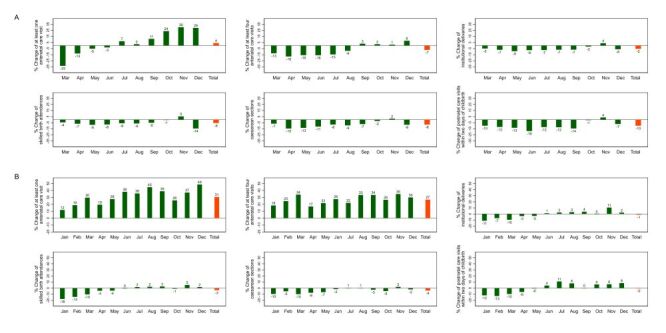
Percent change of essential maternal and child health services, national level, 2020 and 2021. Baseline: average 2018–2019. **Panel A.** 2020. **Panel B.** 2021.

At the national level, there was a reduction in the utilisation of all maternal and child health services in 2020 that was evident primarily between March and August 2020, with a rebound in 2021 (**Figure 1**, panels A–B, Figures S1–S3 in the Online Supplementary Document). Of note, a decreasing number could already be noticed for all services analysed since 2019, except caesarean sections. The annual, quarterly and monthly trends of the number of health services by natural region are shown in Figures S4–S9 in the **Online Supplementary Document**.

All assessed services at the national level showed an annualised percentage decrease of diverse magnitude for 2020 in relation to the baseline period (average of 2018–2019), except for the number of one antenatal care visit or more, which showed a slight annual increase of 4% (**Figure 1**, panel A).

The percent changes in the utilisation of essential maternal and child health services at the national level for 2021, as related to the baseline level (average of 2018–2019) (**Figure 1**, panel B), are shown in Figure S10 in the **Online Supplementary Document**. Two indicators (at least one antenatal care visit and at least four antenatal care visits) showed a clear recovery that went even above pre-pandemic levels. Institutional deliveries and postnatal care visits within two days of childbirth recovered up to pre-pandemic levels, while caesarean sections and skilled birth attendances recovered substantially but without reaching pre-pandemic levels. The rebound of antenatal care visits occurred earlier in September 2020 than the rebound of institutional deliveries, skilled birth attendances and postnatal care, which occurred only after July 2021. There was still an impact on the number of caesarean sections in 2021, though the extent was lower than in 2020.

**Figure 2:**
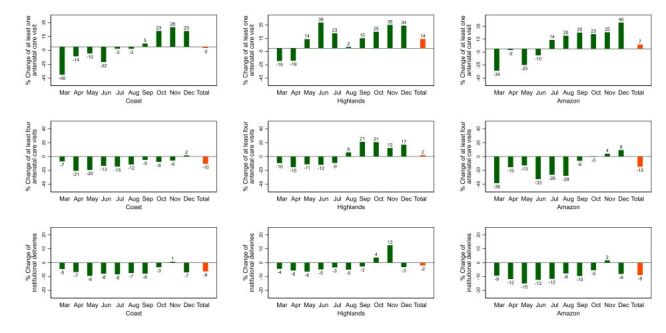
Percent change of essential maternal and child health services by natural region, 2020. Peru. Baseline: average 2018–2019.

**Figure 3:**
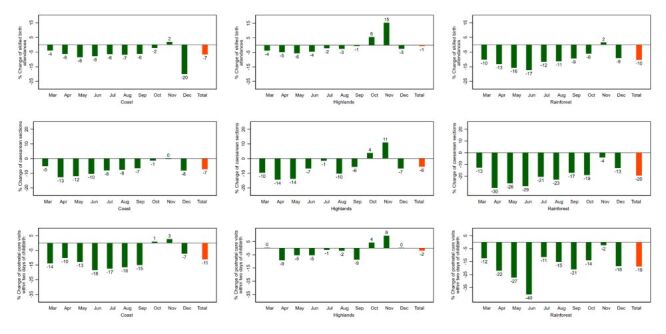
Percent change of essential maternal and child health services by natural region, 2020: Peru. Baseline: average 2018–2019.

**Figure 4:**
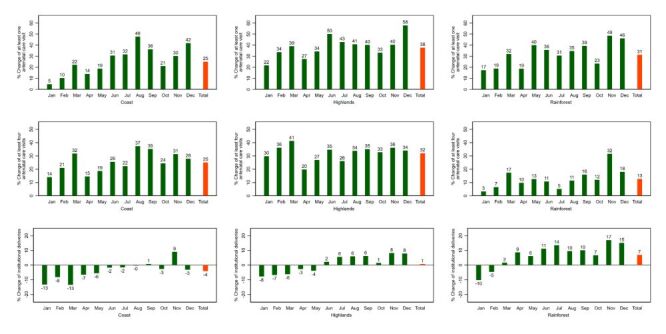
Percent change of essential maternal and child health services by natural region 2021: Peru. Baseline: average 2018–2019.

**Figure 5:**
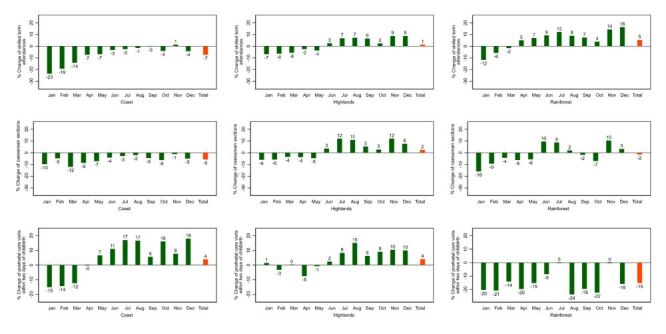
Percent change of essential maternal and child health services by natural region 2021: Peru. Baseline: average 2018–2019.

In terms of changes by natural region, in 2020 the Highlands shows less impact for antenatal care and institutional deliveries, while the Rainforest appears more disrupted (**Figure 2**, **Figure 3**). A similar pattern is observed in 2021 (**Figure 4**, **Figure 5**).

The percentage variations at the departmental level in 2020 and 2021 are shown in Figure S11 in the **Online Supplementary Document**.

The most affected departments in 2020, with 11 or more percentage points reduction in the number of the involved service, were Tacna, with five services affected (at least one antenatal care visit, at least four antenatal care visits, institutional deliveries, skilled birth attendances and caesarean sections); Tumbes, with four services affected (at least one antenatal care visit, at least four antenatal care visits, caesarean sections and postnatal control visits within two days of childbirth); Lima, with three services affected (at least one antenatal care visit; at least four antenatal care visits and postnatal control visits within two days of childbirth), and Loreto, with three services affected (at least four antenatal care visits, caesarean sections and postnatal control visits within two days of childbirth).

The departments most affected in 2021, with 11 or more percentage points reduction of the involved service, were Lima, Arequipa, and Tacna, with five services most affected each (Lima: at least one antenatal care visit, at least four antenatal care visits, institutional deliveries, caesarean sections and postnatal control visits within two days of childbirth; Arequipa: at least one antenatal care visit, at least four antenatal care visits, institutional deliveries, skilled birth attendances and postnatal control visits within two days of childbirth; and Tacna: at least one antenatal care visit, at least four antenatal care visits, institutional deliveries, skilled birth attendances and caesarean sections).

**Figure 6:**
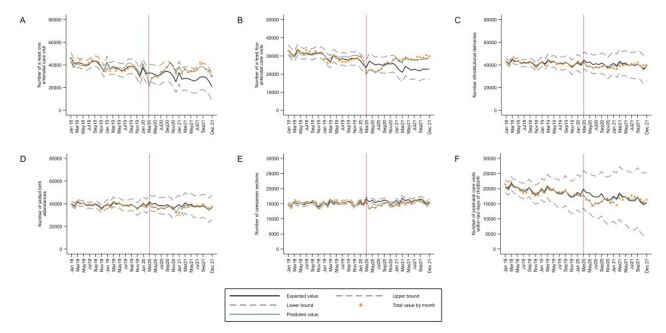
Interrupted time series plots of trends in count of essential maternal and child health services at the national level, Peru: 2018–2021. **Panel A.** At least one antenatal care visit. **Panel B.** At least four antenatal care visits. **Panel C.** Institutional deliveries. **Panel D.** Skilled birth attendances. **Panel E.** Caesarean sections. **Panel F.** Postnatal care visits**.**

**Figure 7:**
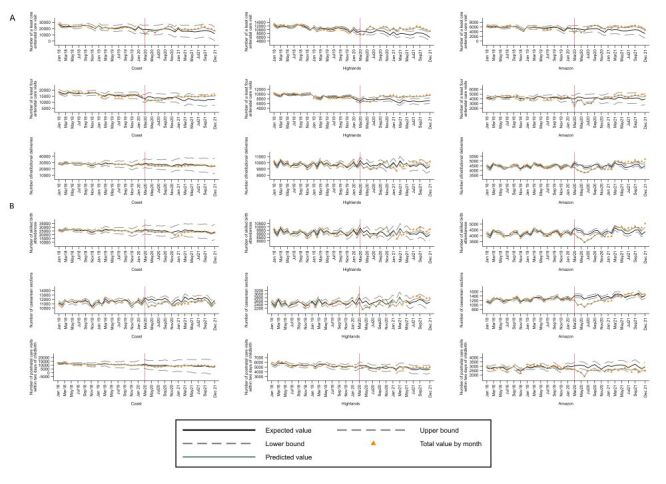
Interrupted time series plots of trends in count of essential maternal and child health services by natural region, Peru: 2018–2021. Interrupted time series plots of trends in count of essential maternal and child health services by natural region, Peru: 2018–2021. **Panel A.** At least one antenatal care visit, at least four antenatal care visits and institutional deliveries. **Panel B.** Skilled birth attendances, caesarean sections and postnatal care visits.

In terms of the interrupted time series analyses, at the national level, during 2020 there was a substantial decrease in the utilisation of all assessed services, including at least one antenatal care visit (incidence rate ratio (IRR) = 0.79; 95% CI = 0.74–0.83), at least four antenatal care visits (IRR = 0.76; CI = 0.74–0.79), institutional deliveries (IRR = 0.94; CI = 0.93–0.96), skilled birth attendances (IRR = 0.95; CI = 0.94–0.97), caesarean sections (IRR = 0.91; CI = 0.89–0.93) and postnatal care visits within two days of childbirth (IRR = 0.87; CI = 0.84–0.90) (**Table 1**, **Figure 6**). In 2021 there was a rebound in the number of all the services that was more evident from July onwards for most of them, with institutional deliveries (IRR = 0.95; CI = 0.93–0.97) and skilled birth attendances (IRR = 0.92; CI = 0.9–0.95) showing a degree of recovery that was substantial but not reaching pre-pandemic levels (**Table 1**, **Figure 6**). Results at the level of natural regions are shown in **Table 2**, **Table 3**, **Table 4**, **Figure 7**, panels A–B.

**Table 1:** Interrupted time series analysis of counts of essential maternal and child health care services at the national level, Peru: 2018–2021

Month	At least one antenatal care visit		At least four antenatal care visits		Institutional deliveries		Skilled birth attendances		Caesarean sections		Postnatal care visits within two days of childbirth	
	**IRR (95% CI)**	***P*-value**	**IRR (95% CI)**	***P*-value**	**IRR (95% CI)**	***P*-value**	**IRR (95% CI)**	***P*-value**	**IRR (95% CI)**	***P*-value**	**IRR (95% CI)**	**P-value**
March 2020	0.60 (0.58–0.61)	<0.001	0.61 (0.59–0.62)	<0.001	0.99 (0.98–0.99)	<0.001	1.00 (0.99–1.00)	0.377	1.01 (1.01–1.02)	<0.001	0.94 (0.92–0.96)	<0.001
April 2020	0.79 (0.76–0.81)	<0.001	0.66 (0.64–0.68)	<0.001	0.91 (0.91–0.92)	<0.001	0.92 (0.91–0.93)	<0.001	0.87 (0.86–0.88)	<0.001	0.87 (0.85–0.88)	<0.001
May 20	0.83 (0.79–0.85)	<0.001	0.65 (0.62–0.68)	<0.001	0.91 (0.90–0.92)	<0.001	0.92 (0.91–0.93)	<0.001	0.90 (0.89–0.91)	<0.001	0.86 (0.84–0.87)	<0.001
June 2020	0.81 (0.78–0.84)	<0.001	0.62 (0.59–0.66)	<0.001	0.88 (0.87–0.89)	<0.001	0.89 (0.88–0.90)	<0.001	0.87 (0.86–0.88)	<0.001	0.77 (0.76–0.79)	<0.001
July 2020	0.92 (0.88–0.96)	<0.001	0.64 (0.6–0.68)	<0.001	0.92 (0.91–0.93)	<0.001	0.93 (0.92–0.94)	<0.001	0.94 (0.93–0.95)	<0.001	0.84 (0.82–0.86)	<0.001
August 2020	0.94 (0.89–0.97)	0.003	0.67 (0.63–0.71)	<0.001	0.92 (0.91–0.93)	<0.001	0.93 (0.92–0.94)	<0.001	0.93 (0.92–0.94)	<0.001	0.86 (0.83–0.88)	<0.001
September 2020	1.03 (0.99–1.07)	0.125	0.75 (0.71–0.79)	<0.001	0.96 (0.95–0.97)	<0.001	0.99 (0.97–1.00)	0.007	0.99 (0.98–1.00)	0.07	0.91 (0.88–0.93)	<0.001
October 2020	1.14 (1.10–1.17)	<0.001	0.77 (0.74–0.81)	<0.001	0.98 (0.98–0.99)	<0.001	1.01 (1.00–1.02)	0.172	1.02 (1.02–1.03)	<0.001	0.97 (0.95–0.99)	<0.001
November 2020	1.10 (1.07–1.12)	<0.001	0.77 (0.75–0.79)	<0.001	0.96 (0.96–0.97)	<0.001	0.98 (0.98–0.99)	<0.001	0.98 (0.97–0.98)	<0.001	0.97 (0.95–0.98)	<0.001
December 2020	0.93 (0.91–0.95)	<0.001	0.85 (0.83–0.86)	<0.001	0.93 (0.93–0.93)	<0.001	0.85 (0.85–0.85)	<0.001	0.94 (0.93–0.94)	<0.001	0.88 (0.87–0.89)	<0.001
Overall 2020	0.79 (0.74–0.83)	<0.001	0.76 (0.74–0.79)	<0.001	0.94 (0.93–0.96)	<0.001	0.95 (0.94–0.97)	<0.001	0.91 (0.89–0.93)	<0.001	0.87 (0.84–0.90)	<0.001
January 2021	1.10 (1.08–1.11)	<0.001	0.90 (0.89–0.91)	<0.001	0.90 (0.90–0.90)	<0.001	0.84 (0.83–0.84)	<0.001	0.96 (0.95–0.96)	<0.001	0.88 (0.87–0.89)	<0.001
February 2021	0.99 (0.98–1.00)	0.205	0.88 (0.87–0.88)	<0.001	0.88 (0.87–0.88)	<0.001	0.81 (0.81–0.82)	<0.001	0.93 (0.93–0.94)	<0.001	0.82 (0.81–0.83)	<0.001
March 2021	1.14 (1.12–1.15)	<0.001	0.91 (0.89–0.92)	<0.001	0.93 (0.93–0.93)	<0.001	0.93 (0.92–0.93)	<0.001	0.98 (0.97–0.98)	<0.001	0.93 (0.92–0.94)	<0.001
April 2021	1.05 (1.03–1.07)	<0.001	0.93 (0.91–0.95)	<0.001	0.94 (0.93–0.94)	<0.001	0.93 (0.93–0.94)	<0.001	0.94 (0.93–0.95)	<0.001	0.91 (0.90–0.92)	<0.001
May 21	1.08 (1.06–1.10)	<0.001	0.93 (0.89–0.96)	<0.001	0.96 (0.95–0.96)	<0.001	0.96 (0.95–0.96)	<0.001	0.97 (0.96–0.98)	<0.001	0.97 (0.96–0.99)	0.003
June 2021	1.12 (1.09–1.14)	<0.001	0.92 (0.88–0.96)	<0.001	0.96 (0.95–0.96)	<0.001	0.95 (0.95–0.96)	<0.001	0.97 (0.96–0.98)	<0.001	0.97 (0.95–1.00)	0.033
July 2021	1.13 (1.10–1.16)	<0.001	0.9 (0.86–0.95)	<0.001	1.00 (0.99–1.01)	0.739	1.00 (0.99–1.01)	0.485	1.03 (1.02–1.04)	<0.001	1.04 (1.01–1.07)	0.017
August 2021	1.31 (1.27–1.34)	<0.001	0.96 (0.91–1.00)	0.068	1.01 (1.00–1.02)	0.03	1.01 (1.00–1.01)	0.188	1.03 (1.02–1.04)	<0.001	1.03 (1.00–1.07)	0.033
September 2021	1.27 (1.24–1.30)	<0.001	0.94 (0.9–0.98)	0.005	1.06 (1.05–1.07)	<0.001	1.06 (1.05–1.06)	<0.001	1.04 (1.03–1.05)	<0.001	1.05 (1.02–1.08)	0.001
October 2021	1.14 (1.11–1.15)	<0.001	0.94 (0.91–0.96)	<0.001	0.99 (0.99–1.00)	<0.001	0.99 (0.99–1.00)	<0.001	1.00 (0.99 -1.00)	0.48	1.01 (0.99–1.03)	0.178
November 2021	1.12 (1.10–1.12)	<0.001	1.00 (0.98–1.01)	0.822	1.01 (1.01–1.01)	<0.001	0.97 (0.97–0.97)	<0.001	0.98 (0.98 -0.99)	<0.001	0.97 (0.96–0.98)	<0.001
December 2021	1.00 (0.00–0.00)	<0.001	1.00 (0.00–0.00)	<0.001	1.00 (0.00–0.00)	<0.001	1.00 (0.00–0.00)	<0.001	1.00 (0.00-0.00)	<0.001	1.00 (0.00–0.00)	<0.001
Overall 2021	1.00 (0.92–1.09)	0.949	1.07 (1.04–1.11)	<0.001	0.95 (0.93–0.97)	<0.001	0.92 (0.9–0.95)	<0.001	0.99 (0.97-1.02)	0.602	0.96 (0.92–1.00)	0.070

CI – confidence interval, IRR – incidence rate ratio

**Table 2:** Interrupted time series analysis of counts of essential maternal and child health care services by natural region, Peru: 2018–2021, Coast

Month	At least one antenatal care visit		At least four antenatal care visits		Institutional deliveries		Skilled birth attendances		Caesarean sections		Postnatal care visits within two days of childbirth	
	**IRR (95% CI)**	***P*-value**	**IRR (95% CI)**	***P*-value**	**IRR (95% CI)**	***P*-value**	**IRR (95% CI)**	***P*-value**	**IRR (95% CI)**	***P*-value**	**IRR (95% CI)**	***P*-value**
March 2020	0.56 (0.55–0.57)	<0.001	0.66 (0.64–0.68)	<0.001	1.04 (1.03–1.05)	<0.001	1.06 (1.05–1.07)	<0.001	1.05 (1.04–1.05)	<0.001	0.91 (0.88–0.93)	<0.001
April 2020	0.82 (0.80–0.84)	<0.001	0.65 (0.63–0.67)	<0.001	0.96 (0.95–0.97)	<0.001	0.98 (0.97–0.99)	<0.001	0.91 (0.90–0.91)	<0.001	0.85 (0.83–0.87)	<0.001
May 20	0.84 (0.81–0.87)	<0.001	0.64 (0.61–0.67)	<0.001	0.95 (0.94–0.96)	<0.001	0.97 (0.96–0.98)	<0.001	0.93 (0.92–0.94)	<0.001	0.83 (0.81–0.84)	<0.001
June 2020	0.70 (0.68–0.72)	<0.001	0.65 (0.62–0.69)	<0.001	0.93 (0.91–0.94)	<0.001	0.94 (0.93–0.95)	<0.001	0.91 (0.90–0.92)	<0.001	0.74 (0.73–0.76)	<0.001
July 2020	0.89 (0.86–0.92)	<0.001	0.66 (0.61–0.70)	<0.001	0.96 (0.95–0.97)	<0.001	0.99 (0.97–1.00)	0.064	0.97 (0.96–0.98)	<0.001	0.78 (0.76–0.81)	<0.001
August 2020	0.94 (0.90–0.97)	0.003	0.66 (0.62–0.70)	<0.001	0.97 (0.95–0.98)	<0.001	0.98 (0.97–1.00)	0.027	0.97 (0.96–0.98)	<0.001	0.80 (0.78–0.83)	<0.001
September 2020	1.03 (1.00–1.07)	0.125	0.69 (0.65–0.73)	<0.001	1.00 (0.99–1.01)	0.855	1.03 (1.02–1.05)	<0.001	1.02 (1.01–1.03)	0.068	0.89 (0.87–0.91)	<0.001
October 2020	1.19 (1.16–1.23)	<0.001	0.71 (0.68–0.75)	<0.001	1.01 (1.00–1.02)	0.015	1.04 (1.03–1.06)	<0.001	1.05 (1.05–1.06)	<0.001	0.95 (0.93–0.97)	<0.001
November 2020	1.15 (1.13–1.18)	<0.001	0.73 (0.71–0.75)	<0.001	0.97 (0.96–0.98)	<0.001	1.00 (0.99–1.01)	0.697	0.98 (0.98–0.99)	<0.001	0.92 (0.91–0.94)	<0.001
December 2020	0.93 (0.92–0.95)	<0.001	0.81 (0.80–0.83)	<0.001	0.97 (0.97–0.98)	<0.001	0.85 (0.84–0.85)	<0.001	0.97 (0.96–0.97)	<0.001	0.82 (0.81–0.84)	<0.001
Overall 2020	0.71 (0.67–0.75)	<0.001	0.78 (0.75–0.82)	<0.001	0.94 (0.93–0.96)	<0.001	0.97 (0.95–0.99)	0.004	0.92 (0.91–0.95)	<0.001	0.86 (0.82–0.90)	<0.001
January 2021	1.11 (1.10–1.13)	<0.001	0.89 (0.88–0.90)	<0.001	0.92 (0.92–0.93)	<0.001	0.83 (0.82–0.83)	<0.001	0.98 (0.98–0.98)	<0.001	0.82 (0.81–0.84)	<0.001
February 2021	0.97 (0.96–0.98)	0.205	0.86 (0.85–0.87)	<0.001	0.91 (0.91–0.92)	<0.001	0.81 (0.81–0.82)	<0.001	0.93 (0.93–0.94)	<0.001	0.79 (0.78–0.81)	<0.001
March 2021	1.12 (1.10–1.13)	<0.001	0.90 (0.89–0.92)	<0.001	0.93 (0.93–0.94)	<0.001	0.94 (0.93–0.94)	<0.001	0.93 (0.93–0.94)	<0.001	0.91 (0.89–0.93)	<0.001
April 2021	1.05 (1.03–1.07)	<0.001	0.92 (0.90–0.94)	<0.001	0.95 (0.94–0.96)	<0.001	0.96 (0.95–0.96)	<0.001	0.93 (0.93–0.94)	<0.001	0.92 (0.91–0.94)	<0.001
May 21	1.07 (1.05–1.09)	<0.001	0.92 (0.89–0.96)	<0.001	0.98 (0.97–0.99)	<0.001	0.98 (0.97–0.99)	<0.001	0.93 (0.93–0.94)	<0.001	0.99 (0.96–1.01)	0.237
June 2021	1.13 (1.10–1.15)	<0.001	0.92 (0.88–0.97)	0.001	0.98 (0.97–0.99)	<0.001	0.98 (0.97–0.99)	<0.001	0.93 (0.93–0.94)	<0.001	0.98 (0.95–1.01)	0.242
July 2021	1.16 (1.13–1.19)	<0.001	0.91 (0.86–0.96)	0.001	1.02 (1.01–1.03)	<0.001	1.02 (1.01–1.03)	0.002	0.93 (0.93–0.94)	<0.001	1.08 (1.04–1.12)	<0.001
August 2021	1.39 (1.36–1.42)	<0.001	0.99 (0.94–1.05)	0.761	1.03 (1.02–1.04)	<0.001	1.03 (1.02–1.04)	<0.001	0.93 (0.93–0.94)	<0.001	1.08 (1.04–1.13)	<0.001
September 2021	1.32 (1.29–1.34)	<0.001	0.95 (0.91–1.00)	0.034	1.08 (1.07–1.09)	<0.001	1.08 (1.08–1.09)	<0.001	0.93 (0.93–0.94)	<0.001	1.08 (1.04–1.12)	<0.001
October 2021	1.15 (1.14–1.17)	< 0.001	0.94 (0.91-0.97)	<0.001	1.01 (1.00–1.01)	0.032	1.01 (1.00–1.02)	0.003	0.93 (0.93–0.94)	0.523	1.05 (1.03–1.08)	<0.001
November 2021	1.13 (1.12–1.13)	< 0.001	0.99 (0.98-1.01)	0.431	1.03 (1.03–1.04)	<0.001	0.98 (0.97–0.98)	<0.001	0.93 (0.93–0.94)	<0.001	0.93 (0.92–0.94)	<0.001
December 2021	1.00 (0.00–0.00)	< 0.001	1.00 (0.00-0.00)	<0.001	1.00 (0.00–0.00)	<0.001	1.00 (0.00–0.00)	<0.001	0.93 (0.93–0.94)	<0.001	1.00 (0.00–0.00)	<0.001
Overall 2021	0.97 (0.88–1.00)	0.631	1.13 (1.09-1.19)	<0.001	0.95 (0.93–0.97)	<0.001	0.92 (0.89–0.95)	<0.001	0.99 (0.97–1.02)	0.602	0.98 (0.93–1.04)	0.070

CI – confidence interval, IRR – incidence rate ratio

**Table 3:** Interrupted time series analysis of counts of essential maternal and child health care services by natural region, Peru: 2018–2021, Highlands

Month	At least one antenatal care visit		At least four antenatal care visits		Institutional deliveries		Skilled birth attendances		Caesarean sections		Postnatal care visits within two days of childbirth	
	**IRR (95% CI)**	***P*-value**	**IRR (95% CI)**	***P*-value**	**IRR (95% CI)**	***P*-value**	**IRR (95% CI)**	***P*-value**	**IRR (95% CI)**	***P*-value**	**IRR (95% CI)**	***P*-value**
March 2020	0.65 (0.63–0.67)	<0.001	0.57 (0.55–0.59)	<0.001	0.94 (0.93–0.95)	<0.001	0.94 (0.93–0.96)	<0.001	1.05 (1.04–1.05)	<0.001	0.96 (0.93–0.96)	<0.001
April 2020	0.65 (0.63–0.67)	<0.001	0.64 (0.61–0.66)	<0.001	0.87 (0.86–0.88)	<0.001	0.87 (0.86–0.89)	<0.001	0.91 (0.90–0.91)	<0.001	0.86 (0.84–0.86)	<0.001
May 20	0.88 (0.85–0.92)	<0.001	0.62 (0.60–0.65)	<0.001	0.89 (0.88–0.91)	<0.001	0.90 (0.89–0.92)	<0.001	0.93 (0.92–0.94)	<0.001	0.91 (0.88–0.91)	<0.001
June 2020	1.01 (0.97–1.06)	0.565	0.60 (0.57–0.63)	<0.001	0.86 (0.85–0.87)	<0.001	0.87 (0.85–0.88)	<0.001	0.91 (0.90–0.92)	<0.001	0.86 (0.84–0.86)	<0.001
July 2020	0.92 (0.87–0.97)	0.001	0.63 (0.60–0.67)	<0.001	0.90 (0.89–0.91)	<0.001	0.91 (0.89–0.92)	<0.001	0.97 (0.96–0.98)	<0.001	0.88 (0.85–0.88)	<0.001
August 2020	0.84 (0.79–0.88)	<0.001	0.71 (0.67–0.75)	<0.001	0.88 (0.88–0.89)	<0.001	0.90 (0.89–0.92)	<0.001	0.97 (0.96–0.98)	<0.001	0.88 (0.85–0.88)	<0.001
September 2020	0.93 (0.88–0.97)	0.003	0.80 (0.76–0.84)	<0.001	0.95 (0.94–0.96)	<0.001	0.97 (0.95–0.98)	<0.001	1.02 (1.01–1.03)	<0.001	0.87 (0.85–0.87)	<0.001
October 2020	1.00 (0.96–1.04)	0.948	0.82 (0.80–0.86)	<0.001	0.98 (0.98–0.99)	<0.001	1.00 (0.99–1.01)	0.885	1.05 (1.05–1.06)	0.885	0.97 (0.95–0.97)	<0.001
November 2020	1.02 (0.98–1.05)	0.339	0.80 (0.78–0.82)	<0.001	1.01 (1.00–1.02)	0.002	1.03 (1.02–1.04)	<0.001	0.98 (0.98–0.99)	<0.001	0.99 (0.98–0.99)	<0.001
December 2020	0.89 (0.87–0.91)	<0.001	0.87 (0.85–0.88)	<0.001	0.90 (0.89–0.9)	<0.001	0.90 (0.89–0.91)	<0.001	0.97 (0.96–0.97)	<0.001	0.93 (0.93–0.93)	<0.001
Overall 2020	0.93 (0.87–1.00)	0.053	0.79 (0.77–0.80)	<0.001	0.96 (0.94–0.97)	<0.001	0.96 (0.95–0.98)	<0.001	0.91 (0.89–0.93)	**<0.001**	**0.96 (0.94–0.98)**	**0.001**
January 2021	1.04 (1.03–1.06)	<0.001	0.91 (0.90–0.92)	<0.001	0.90 (0.89–0.90)	<0.001	0.90 (0.89–0.91)	<0.001	0.98 (0.98–0.98)	<0.001	0.93 (0.91–0.93)	<0.001
February 2021	0.97 (0.96–0.98)	<0.001	0.90 (0.89–0.91)	<0.001	0.84 (0.83–0.84)	<0.001	0.84 (0.83–0.84)	<0.001	0.93 (0.93–0.94)	<0.001	0.81 (0.80–0.81)	<0.001
March 2021	1.12 (1.11–1.14)	<0.001	0.88 (0.87–0.90)	<0.001	0.93 (0.92–0.93)	<0.001	0.93 (0.92–0.93)	<0.001	0.93 (0.93–0.94)	<0.001	0.94 (0.92–0.94)	<0.001
April 2021	1.02 (1.01–1.04)	0.006	0.91 (0.89–0.93)	<0.001	0.90 (0.89–0.91)	<0.001	0.89 (0.89–0.90)	<0.001	0.93 (0.93–0.94)	<0.001	0.87 (0.85–0.87)	<0.001
May 21	1.04 (1.01–1.06)	0.002	0.90 (0.87–0.93)	<0.001	0.92 (0.91–0.93)	<0.001	0.92 (0.91–0.93)	<0.001	0.93 (0.93–0.94)	<0.001	0.93 (0.91–0.93)	0.237
June 2021	1.08 (1.05–1.12)	<0.001	0.93 (0.89–0.96)	0.001	0.93 (0.92–0.94)	<0.001	0.93 (0.92–0.94)	<0.001	0.93 (0.93–0.94)	<0.001	0.91 (0.89–0.91)	0.242
July 2021	1.06 (1.03–1.10)	<0.001	0.89 (0.85–0.92)	0.001	0.99 (0.98–1.00)	0.011	0.99 (0.98–1.00)	0.11	0.93 (0.93–0.94)	0.11	0.94 (0.91–0.94)	<0.001
August 2021	1.16 (1.13–1.20)	<0.001	0.90 (0.86–0.94)	0.761	0.99 (0.98–1.00)	0.003	0.99 (0.98–1.00)	0.159	0.93 (0.93–0.94)	0.159	1.01 (0.98–1.01)	<0.001
September 2021	1.14 (1.10–1.17)	<0.001	0.90 (0.87–0.93)	0.034	1.04 (1.03–1.04)	<0.001	1.03 (1.03–1.04)	<0.001	0.93 (0.93–0.94)	<0.001	0.99 (0.97–0.99)	<0.001
October 2021	1.07 (1.04–1.09)	<0.001	0.91 (0.89–0.93)	<0.001	0.96 (0.96–0.97)	<0.001	0.97 (0.96–0.97)	<0.001	0.93 (0.93–0.94)	<0.001	0.99 (0.97–0.99)	<0.001
November 2021	1.04 (1.03–1.05)	<0.001	0.98 (0.97–0.99)	0.431	0.97 (0.97–0.97)	<0.001	0.97 (0.97–0.97)	<0.001	0.93 (0.93–0.94)	<0.001	0.97 (0.96–0.97)	<0.001
December 2021	1.00 (0.00–0.00)	<0.001	1.00 (0.00–0.00)	<0.001	1.00 (0.00–0.00)	<0.001	1.00 (0.00–0.00)	<0.001	0.93 (0.93–0.94)	<0.001	1.00 (0.00–0.00)	<0.001
Overall 2021	1.04 (0.98–1.09)	0.184	1.01 (0.99–1.04)	0.258	0.95 (0.93–0.97)	<0.001	0.91 (0.89–0.98)	<0.001	0.98 (0.94–1.03)	**0.602**	**0.92 (0.90–0.95)**	**<0.001**

CI - confidence interval, IRR - incidence rate ratio

**Table 4:** Interrupted time series analysis of counts of essential maternal and child health care services by natural region, Peru: 2018–2021, Rainforest

Month	At least one antenatal care visit		At least four antenatal care visits		Institutional deliveries		Skilled birth attendances		Caesarean sections		Postnatal care visits within two days of childbirth	
	**IRR (95% CI)**	***P*-value**	**IRR (95% CI)**	***P*-value**	**IRR (95% CI)**	***P*-value**	**IRR (95% CI)**	***P*-value**	**IRR (95% CI)**	***P*-value**	**IRR (95% CI)**	***P*-value**
March 2020	0.64 (0.62–0.67)	<0.001	0.50 (0.48–0.51)	<0.001	0.83 (0.82–0.84)	<0.001	0.82 (0.81–0.83)	<0.001	0.90 (0.89–0.91)	<0.001	1.09 (1.05–1.13)	<0.001
April 2020	0.94 (0.90–0.98)	0.007	0.75 (0.72–0.78)	<0.001	0.77 (0.76–0.78)	<0.001	0.76 (0.75–0.77)	<0.001	0.72 (0.71–0.73)	<0.001	0.98 (0.94–1.02)	0.232
May 20	0.67 (0.64–0.71)	<0.001	0.76 (0.72–0.80)	<0.001	0.75 (0.74–0.76)	<0.001	0.74 (0.73–0.75)	<0.001	0.74 (0.73–0.75)	<0.001	0.91 (0.87–0.95)	<0.001
June 2020	0.78 (0.74–0.83)	<0.001	0.55 (0.52–0.59)	<0.001	0.73 (0.72–0.74)	<0.001	0.69 (0.68–0.70)	<0.001	0.65 (0.64–0.66)	<0.001	0.74 (0.70–0.78)	<0.001
July 2020	1.04 (0.98–1.10)	0.173	0.62 (0.58–0.66)	<0.001	0.74 (0.73–0.75)	<0.001	0.73 (0.72–0.75)	<0.001	0.75 (0.74–0.76)	<0.001	1.02 (0.96–1.08)	0.474
August 2020	1.16 (1.10–1.22)	<0.001	0.61 (0.57–0.65)	<0.001	0.80 (0.79–0.81)	<0.001	0.76 (0.75–0.78)	<0.001	0.75 (0.75–0.76)	<0.001	1.07 (1.01–1.14)	0.026
September 2020	1.25 (1.19–1.30)	<0.001	0.81 (0.76–0.86)	<0.001	0.82 (0.80–0.83)	<0.001	0.82 (0.80–0.83)	<0.001	0.83 (0.82–0.84)	<0.001	1.07 (1.01–1.14)	0.019
October 2020	1.23 (1.19–1.27)	<0.001	0.87 (0.84–0.91)	<0.001	0.85 (0.84–0.86)	<0.001	0.85 (0.84–0.86)	<0.001	0.86 (0.85–0.87)	<0.001	1.07 (1.02–1.13)	0.01
November 2020	1.08 (1.05–1.11)	<0.001	0.84 (0.81–0.86)	<0.001	0.83 (0.82–0.84)	0.002	0.83 (0.82–0.84)	<0.001	0.89 (0.87–0.90)	<0.001	1.11 (1.07–1.15)	<0.001
December 2020	1.02 (1.00–1.04)	0.02	0.92 (0.91–0.94)	<0.001	0.79 (0.79–0.80)	<0.001	0.78 (0.77–0.79)	<0.001	0.84 (0.83–0.85)	<0.001	1.00 (0.97–1.02)	0.857
Overall 2020	0.80 (0.74–0.87)	<0.001	0.65 (0.62–0.68)	<0.001	0.88 (0.84–0.91)	<0.001	0.86 (0.82–0.90)	<0.001	0.75 (0.70–0.80)	**<0.001**	**0.76 (0.71–0.81)**	**0.001**
January 2021	1.18 (1.16–1.19)	<0.001	0.91 (0.90–0.92)	<0.001	0.79 (0.79–0.80)	<0.001	0.77 (0.77–0.78)	<0.001	0.83 (0.82–0.84)	<0.001	1.01 (0.99–1.02)	0.255
February 2021	1.10 (1.09–1.12)	<0.001	0.90 (0.89–0.90)	<0.001	0.79 (0.78–0.80)	<0.001	0.77 (0.77–0.78)	<0.001	0.84 (0.84–0.85)	<0.001	0.94 (0.93–0.96)	<0.001
March 2021	1.27 (1.24–1.30)	<0.001	0.96 (0.94–0.97)	<0.001	0.94 (0.93–0.94)	<0.001	0.90 (0.89–0.91)	<0.001	0.98 (0.97–0.99)	0.002	1.04 (1.01–1.06)	0.003
April 2021	1.13 (1.09–1.16)	<0.001	0.98 (0.96–1.00)	0.087	0.96 (0.95–0.97)	<0.001	0.92 (0.92–0.93)	<0.001	0.97 (0.95–0.98)	<0.001	0.98 (0.95–1.00)	0.101
May 21	1.23 (1.18–1.28)	<0.001	0.98 (0.95–1.02)	0.400	0.94 (0.93–0.95)	<0.001	0.94 (0.93–0.95)	<0.001	0.95 (0.93–0.97)	<0.001	1.03 (1.00–1.07)	0.073
June 2021	1.15 (1.10–1.21)	<0.001	0.92 (0.87–0.96)	<0.001	0.93 (0.92–0.94)	<0.001	0.91 (0.90–0.92)	<0.001	1.00 (0.98–1.02)	0.892	1.09 (1.05–1.14)	<0.001
July 2021	1.17 (1.12–1.23)	<0.001	0.88 (0.84–0.93)	<0.001	0.96 (0.95–0.97)	<0.001	0.93 (0.93–0.94)	<0.001	1.03 (1.01–1.05)	0.002	1.12 (1.08–1.17)	<0.001
August 2021	1.28 (1.23–1.33)	<0.001	0.95 (0.90–1.00)	0.034	0.95 (0.94–0.96)	<0.001	0.94 (0.93–0.95)	<0.001	1.00 (0.98–1.01)	0.794	0.94 (0.90–0.98)	0.002
September 2021	1.37 (1.33–1.42)	<0.001	1.01 (0.97–1.05)	0.688	1.00 (0.99–1.01)	0.542	0.97 (0.96–0.98)	<0.001	0.98 (0.97–0.99)	0.002	1.06 (1.02–1.10)	0.002
October 2021	1.22 (1.20–1.25)	<0.001	0.99 (0.96–1.02)	0.495	0.97 (0.96–0.97)	<0.001	0.94 (0.94–0.95)	<0.001	0.98 (0.97–0.99)	<0.001	0.94 (0.91–0.96)	<0.001
November 2021	1.24 (1.22–1.25)	<0.001	1.06 (1.05–1.08)	<0.001	0.96 (0.96–0.97)	<0.001	0.94 (0.93–0.94)	<0.001	1.02 (1.02–1.03)	<0.001	1.10 (1.09–1.12)	<0.001
December 2021	1.00 (0.00–0.00)	<0.001	1.00 (0.00–0.00)	<0.001	1.00 (0.00–0.00)	<0.001	1.00 (0.00–0.00)	<0.001	1.00 (0.00–0.00)	<0.001	1.00 (0.00–0.00)	<0.001
Overall 2021	1.02 (0.91–1.15)	0.681	0.99 (0.96–1.03)	0.737	1.01 (0.97–1.05)	0.52	0.99 (0.96–1.04)	0.85	1.03 (0.98–1.09)	**0.196**	**0.95 (0.91–1.01)**	**0.137**

CI – confidence interval, IRR – incidence rate ratio

In 2020 the utilisation of all the assessed maternal and child health services decreased in the Coast, being at least one antenatal care visit and at least four antenatal care visits the most affected, while institutional deliveries, skilled birth attendances, caesarean sections, and postnatal care visits within two days of childbirth were affected in a lower degree. In the Highlands, all the services also decreased, with at least four antenatal care visits being the most affected, while the others were comparatively less impacted. As for the Rainforest, all the services were comparatively more affected there than in the other natural regions.

In 2021 the utilisation of most services recovered in all three natural regions, except for skilled birth attendance in the Coast, and institutional deliveries, skilled birth attendance and postnatal care within two days of childbirth in the Highlands that remained at lower levels than in the pre-pandemic period (**Tables 2**, **Table 3**, **Table 4**).

**Figure 8:**
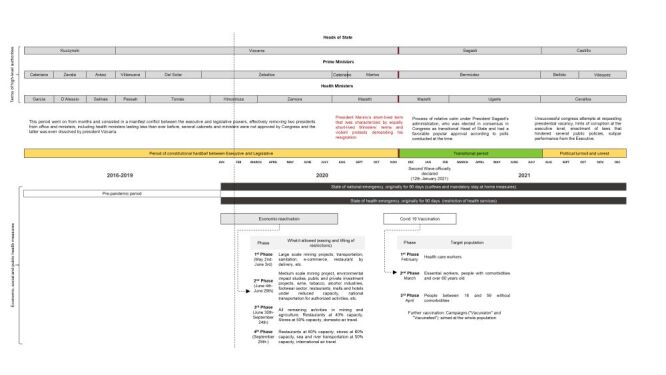
Timeline of the Peruvian political context and of COVID-19 control measures.

The timeline of the Peruvian political context and of the COVID-19 control measures implemented by the government is shown in **Figure 8**. At the time of the onset of the COVID-19 pandemic Peru was facing a political unrest period that had started in 2016 and resulted in six presidents of the republic in office and 16 ministers of health from 2016 to 2022. Also, most high- and intermediate-level officers of the central and regional levels had been removed following the resignment of each minister. The first COVID-19 cases were reported in Peru on early March 2020. The government declared the state of health emergency on 11 March 2020 and the state of national emergency on 15 March 2020, imposing severe restrictions to the movement of people and several restrictions to the delivery of public services, amongst them essential maternal and child health services. Then a progressive easing of restrictions was implemented since early May 2020, with the main objective of reactivating the economy. The COVID-19 vaccination process started in February 2021 and was expanded progressively to different segments of the population. A second wave of COVID-19 was declared on 12 January 2021 that lasted until June 2021.

## DISCUSSION

We found that all the assessed maternal and child health services decreased substantially in Peru in 2020, after the governmental implementation of restrictions aimed at limiting increases in COVID-19 cases. This occurred at national and subnational levels, with a higher impact in the Rainforest than in the Coast and the Highlands. At departmental level the most affected were three coastal departments (Tumbes, Tacna, Lima). Although there was a substantial rebound in 2021 at national and subnational levels, the departments showing still a decrease as compared to the pre-pandemic period were Lima, Arequipa and Tacna, all coastal.

The interpretation of our results needs to be made within the wider political context characterising Peru since 2016. Such a scenario very likely shaped the situation of the Peruvian public sector in general and of the public health system in particular, and thus its capability of providing on a continuous basis public health services, even before the arrival of COVID-19, which could explain the decreasing trend of maternal and child health services already present by 2019. Furthermore, the political crisis characterising Peru since 2016 may have affected the resilience of the public health system and its readiness to continue providing essential maternal and child health services upon the arrival of an emergency situation like the COVID-19 pandemic [17,18]. There is evidence showing that the political instability and the frequent changes in health leadership in Peru have limited the sustainable development of health policies with the consequent inefficiencies and restrictive budgeting laws that in turn led to low investment in health and limited fiscal space [18], which combined with lack of planning and management capabilities, has resulted in low-quality health expenditure and low budget execution [19].

This scenario of political instability compounded the fragmentation that characterises the Peruvian health system, aggravating chronic deficits in infrastructure and in specialised health personnel, as well as weakening more the leadership from health authorities [20,21].

We also need to take into account the timeline of the COVID-19 waves in Peru and the government measures to face them. The first confirmed case occurred on 6 March 2020, and a week later the government imposed a strict lockdown and other severe restrictions [20]. Then a first wave occurred between April 2020 and November, 2020, followed by a devastating second wave that ranged from January to June, 2021, with more than 980 485 reported cases and 98 837 confirmed deaths [22]. The mobility restrictions imposed as a response to the first wave were more severe, while the second wave was faced with much less stringent measures and concurrent efforts to reactivate the economy, which may explain at least in part the rebound in the utilisation of the assessed maternal and child health services.

The decrease in the utilisation of maternal and child health services seen in 2020 may be due to a decreased offer of maternal and child health services, to the diversion of resources to face the pandemic challenges, or to a decreased demand by fear of contagion, as other studies from different regions and countries have proposed [1–6]. In Peru, the decrease is most likely explained by a combination of factors, that is, an already decreased capacity of the health system to provide essential services aggravated by the political crisis described above, a lower health system resilience to respond effectively to exceptional events like the COVID-19 pandemic, the diversion strategies implemented to contain the pandemic, which affected additionally the infrastructure and the resources needed to continue providing maternal and child health services, and finally the decreased population demand for services. For instance, early during the pandemic the government ordered that a substantial proportion of emergency wards, outpatient and inpatient resources of health facilities including health staff and equipment, be diverted to face the demands of COVID-19 patients [23]. Moreover, the health facilities of the primary care level were closed temporarily after the national health emergency was declared, and had reopened only gradually by 2022 [23]. The campaigns of vaccination were also suspended temporarily at national level [23]. These measures affected the continuity of essential maternal and child health services.

In terms of decreased demand-side factors that could have contributed to the reduction in the utilisation of maternal and child health services, fear of contagion, perception that health services would not be available, lack of money to pay for the transportation or for the health services provision, loss of employment or salary, threat of prosecution for leaving home, and distance to health facilities may all have been relevant factors, and have been highlighted in previous reports [24,25]. Future studies are needed to document the relative importance of such factors.

As for factors explaining the rebound in service utilisation seen in our analyses, possible candidates include the easing of the restrictions following subsequent pandemic waves that occurred in Peru after the first two waves and the introduction and expansion of COVID-19 vaccination. As mentioned above, the first COVID-19 wave occurred in Peru in March 2020 and a second wave was declared on 12 January 2021, lasting until June 2021, with less severe restrictions imposed that may have contributed to the rebound by increasing either the supply or the demand for maternal and child health services or both. Likewise, the COVID-19 vaccination process, which started in February 2021, was expanded progressively and very likely contributed to the recovery in the demand of maternal and child health services by reducing the fear of contagion.

The Rainforest was the most affected natural region, while the least affected was the Highlands. This may be due to the fact that the Rainforest is the region that concentrates the poorest and most rural departments, with a greater fragility of its health system in terms of infrastructure, equipment and human resources, which therefore may have been largely surpassed by the pandemic challenges, more than in the Coast and in the Highlands. Besides, historically the rural areas of the Rainforest have shown the lowest coverage figures of maternal and child health services [7].

When we disaggregated the analysis to the departmental level, the most affected departments were those that are coastal, more urbanised and better-off. However, our departmental averages may be concealing inequalities in terms of varying levels of resilience in the health system within each department, including differing degrees of strength in infrastructure and human resources [26].

Strengths of our study include firstly, the fact that we used routinely collected data at the local level and consolidated at regional and central levels. Secondly, we took into account the contextual situation in Peru before the pandemic and after the outbreak. Thirdly, we used a robust regression method adjusted by autocorrelation and seasonality and were able to identify accurately the timeline of the events including the Covid-19 pandemic. Also, our study cover health services through the continuum of maternal and child health care. Finally, we disaggregated the analysis at the subnational level, which allows to take lessons that can be useful for the national and regional levels.

On the other hand, we must acknowledge certain limitations of the study. First, we cannot rule out problems in the completeness and quality of reporting at the local and central levels due to issues in the availability of qualified health officers in charge of the registration and reporting process, although we made efforts to reach consistency through data triangulation with different experts familiar with the administrative health information system. Second, the limited number of interviews may not have captured all the possible perspectives needed to understand the impact of COVID-19 on maternal and child health services, although we carefully selected individuals from diverse sectors. Also, we cannot rule out underreporting or overreporting, although we explored thoroughly the dataset for outliers and reporting completeness. Finally, we could not quantify systematically the consequences of the political crisis affecting Peru since 2016 on the human resources deployment at different levels and on the availability of equipment and supplies, although we were able to obtain the testimony of several officers of the public sector who consistently reported a decline of the health system capacity to provide maternal and child health services.

The COVID-19 pandemic impacted substantially the utilisation of essential maternal and child health services in Peru at national and subnational levels, acting upon a previously weakened public sector. This highlights the need to preserve the resilience of a health system both at central and local levels, so as to face more successfully the challenges posed by a future crisis like the COVID-19 pandemic. On the supply-side there is the urgent need to be prepared to address promptly the infrastructure, workforce, equipment and supplies needs posed by pandemics and other unexpected events, while maintaing the continuity of essential maternal and child health services. On the demand-side, educational efforts are needed to strengthen people awareness about the importance of asking for an uninterrupted provision of essential health services when facing a pandemic or similar event.

## CONCLUSIONS

In conclusion, the lessons learned are useful for central and local health systems to better answer the challenges posed by future pandemics and similar events.

## Additional material


Online Supplementary Document
