# Supplementary material for: Impact of COVID-19 on the utilisation of maternal and child health services in Peru at national and subnational levels: An interrupted time series analysis
Source: J Glob Health. 2024 Dec 20;14:05039. doi: 10.7189/jogh.14.05039 (PMC11659789; doi:10.7189/jogh.14.05039)
Supplement: Online Supplementary Document [file jogh-14-05039-s001.pdf]

Supplementary files

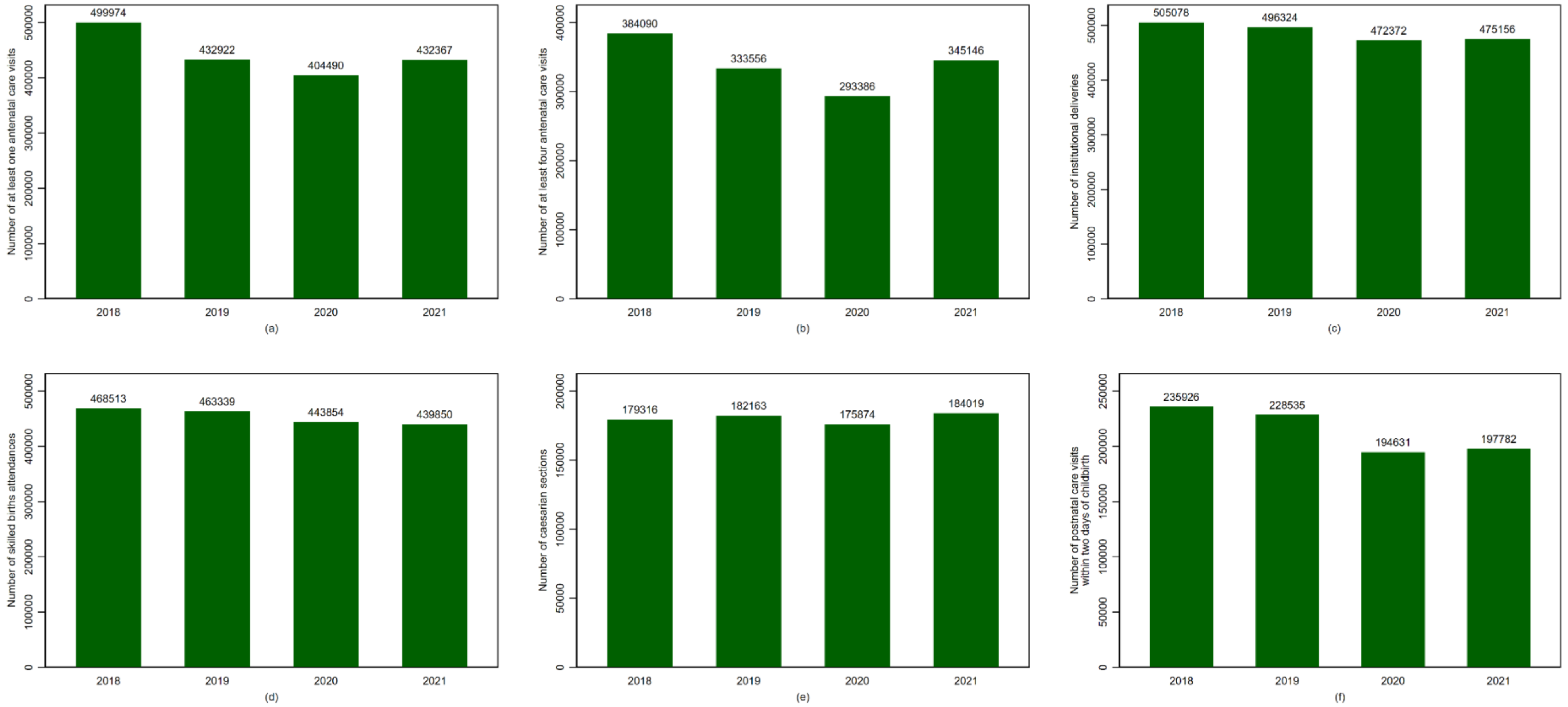

Figure S1. Annual national trend of essential maternal and child health services, 2018-2021.

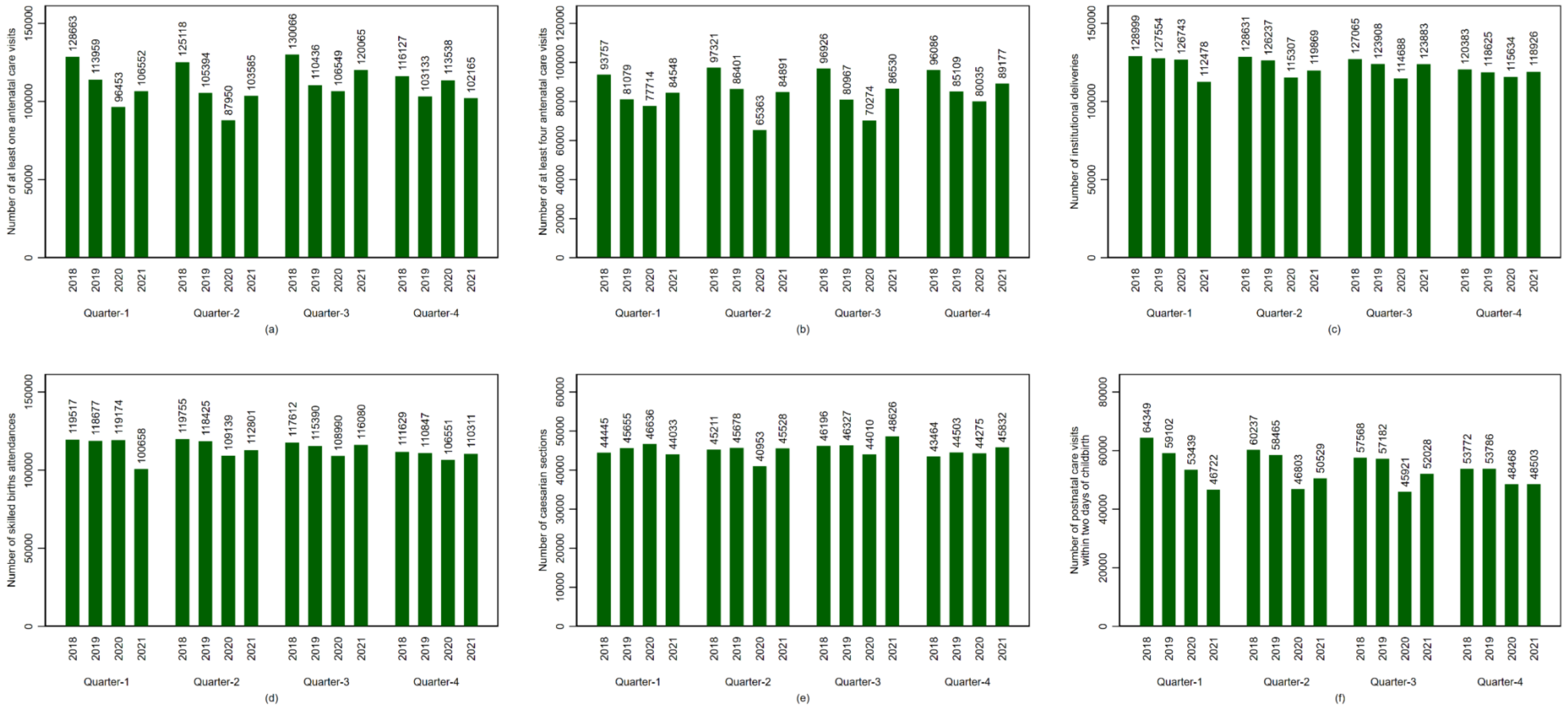

Figure S2. Quarterly national trend of essential maternal and child health services, 2018-2021: Peru.

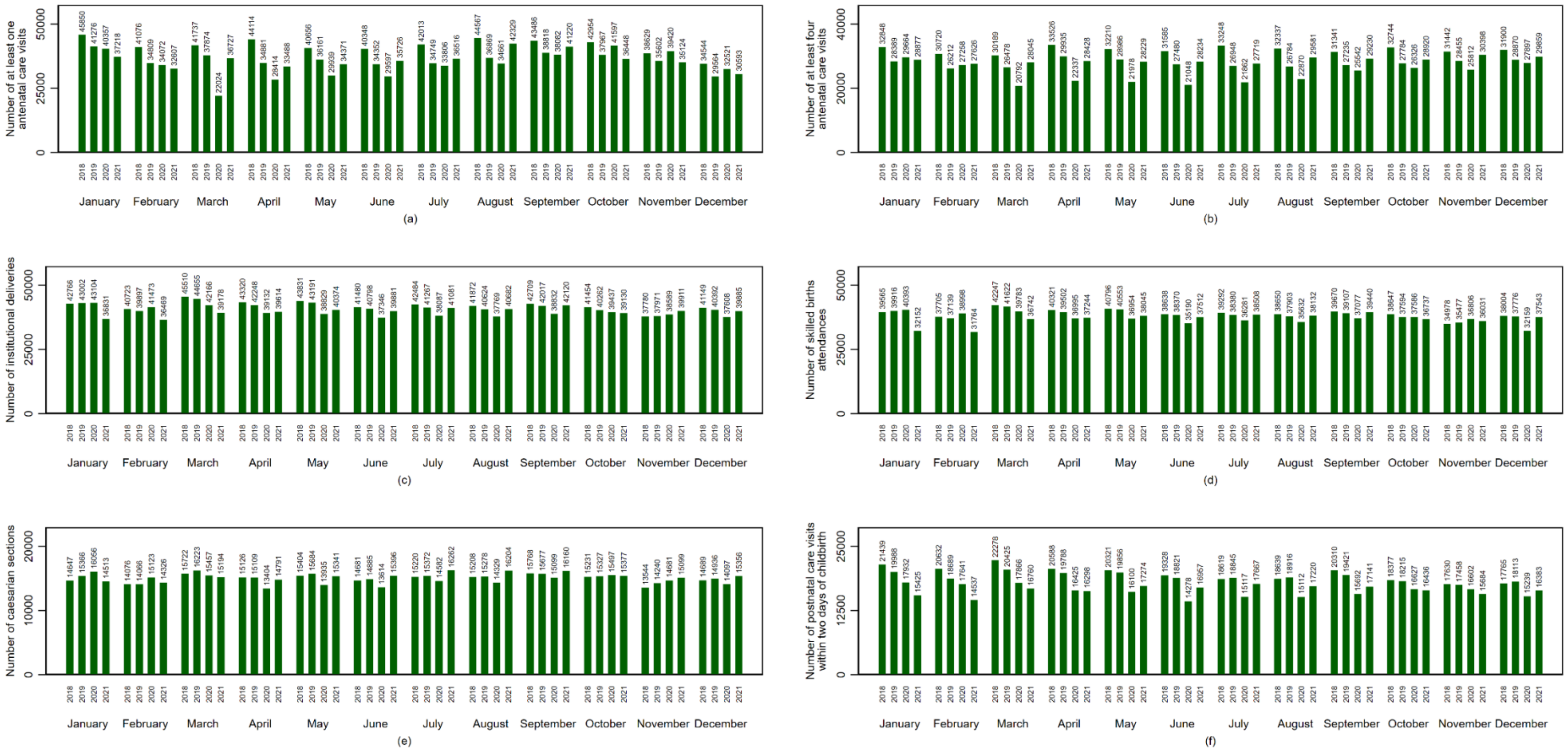

Figure S3. Monthly national trend of essential maternal and child health services, 2018-2021.

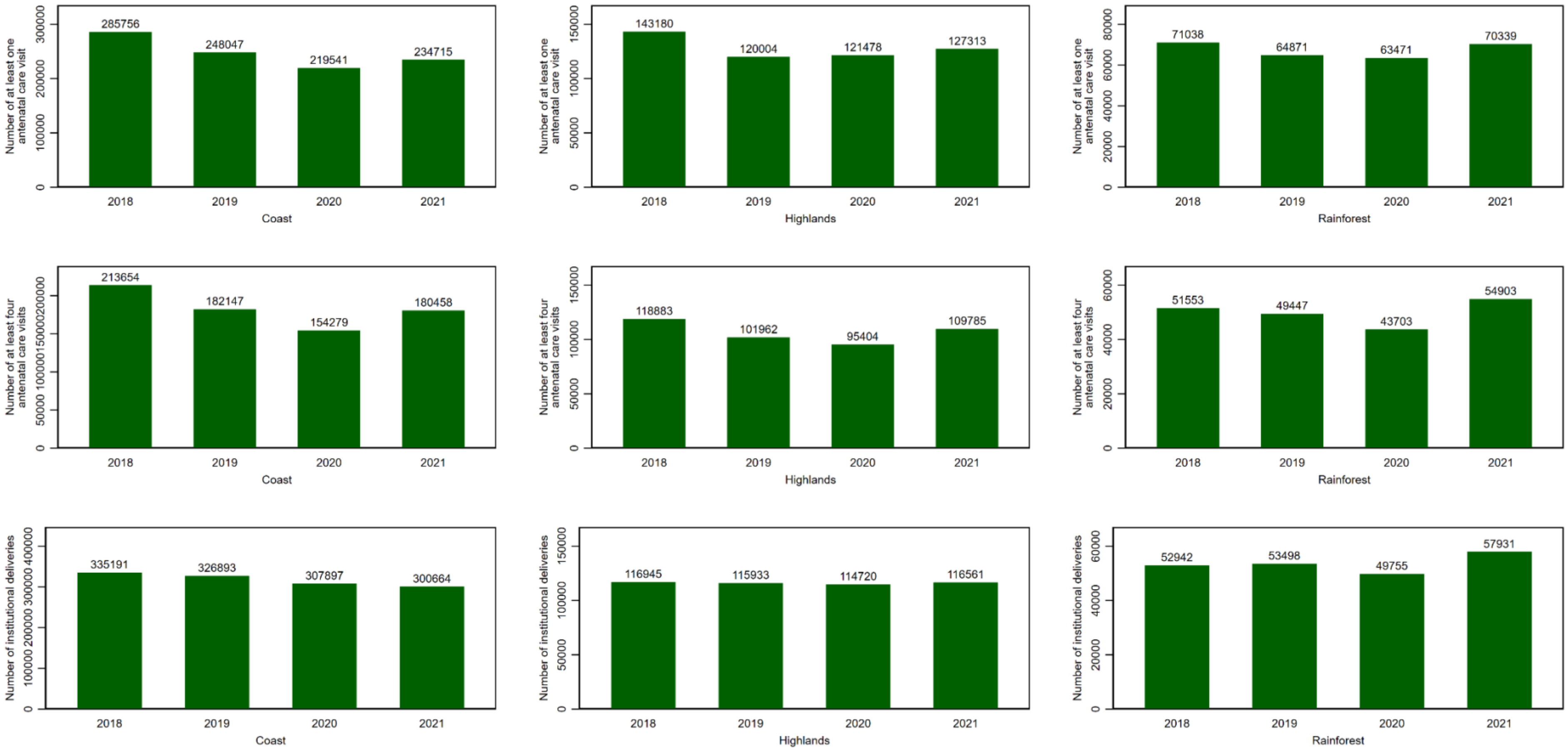

Figure S4. Annual trend of essential maternal and child health services by natural region, 2018-2021: Peru.

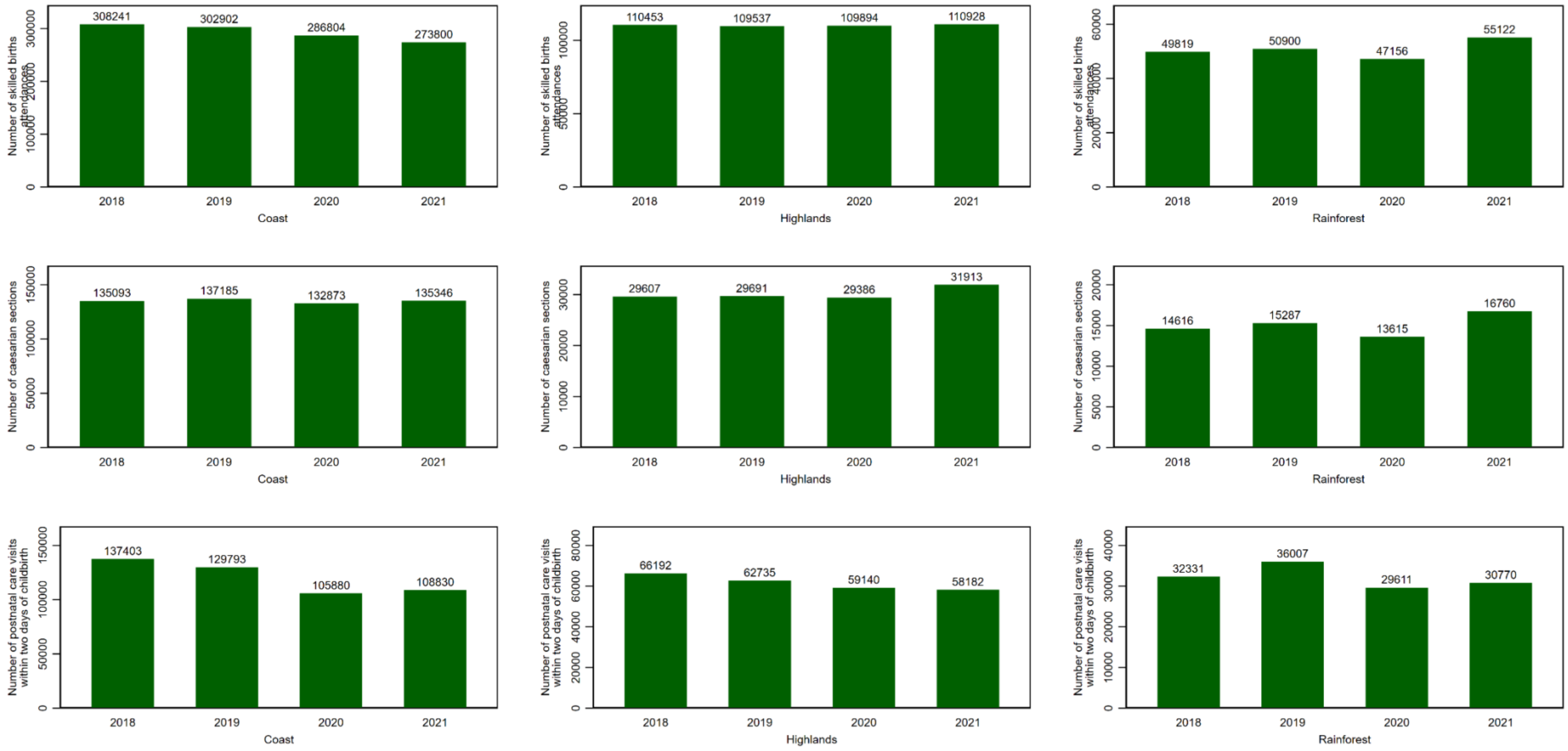

Figure S5. Annual trend of essential maternal and child health services by natural region, 2018-2021: Peru.

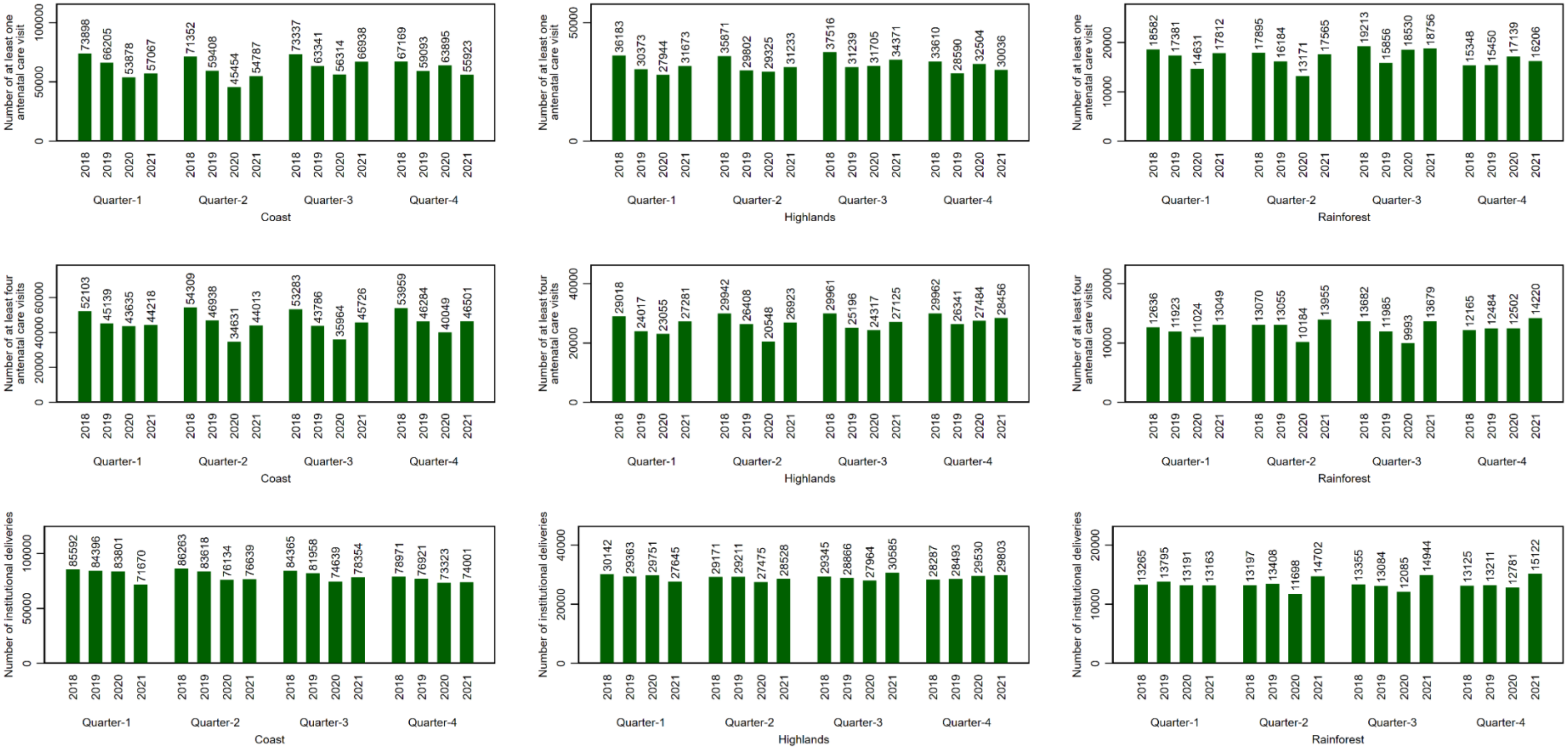

Figure S6. Quarterly trend of essential maternal and child health services by natural region, 2018-2021: Peru.

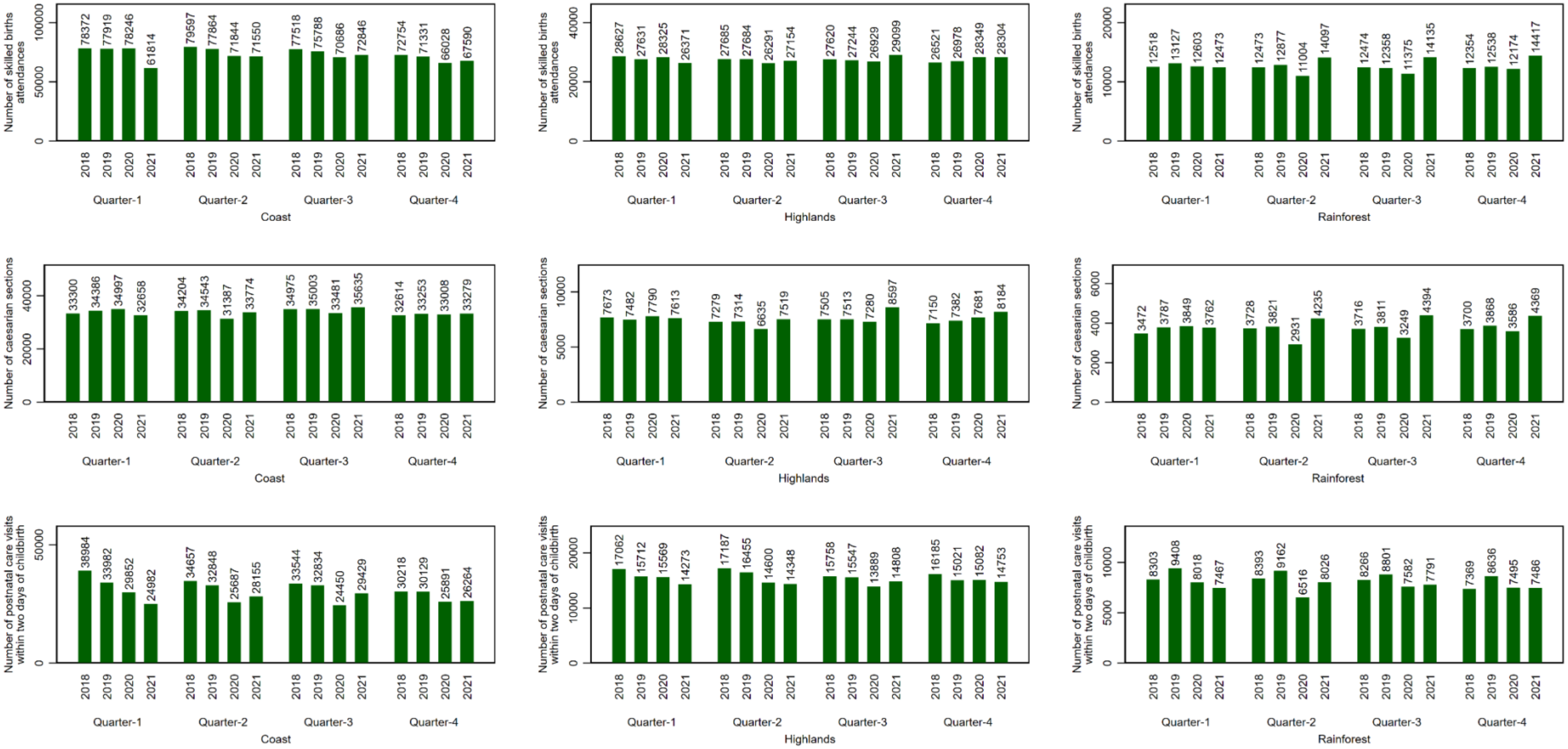

Figure S7. Quarterly trend of essential maternal and child health services by natural region, 2018-2021: Peru.

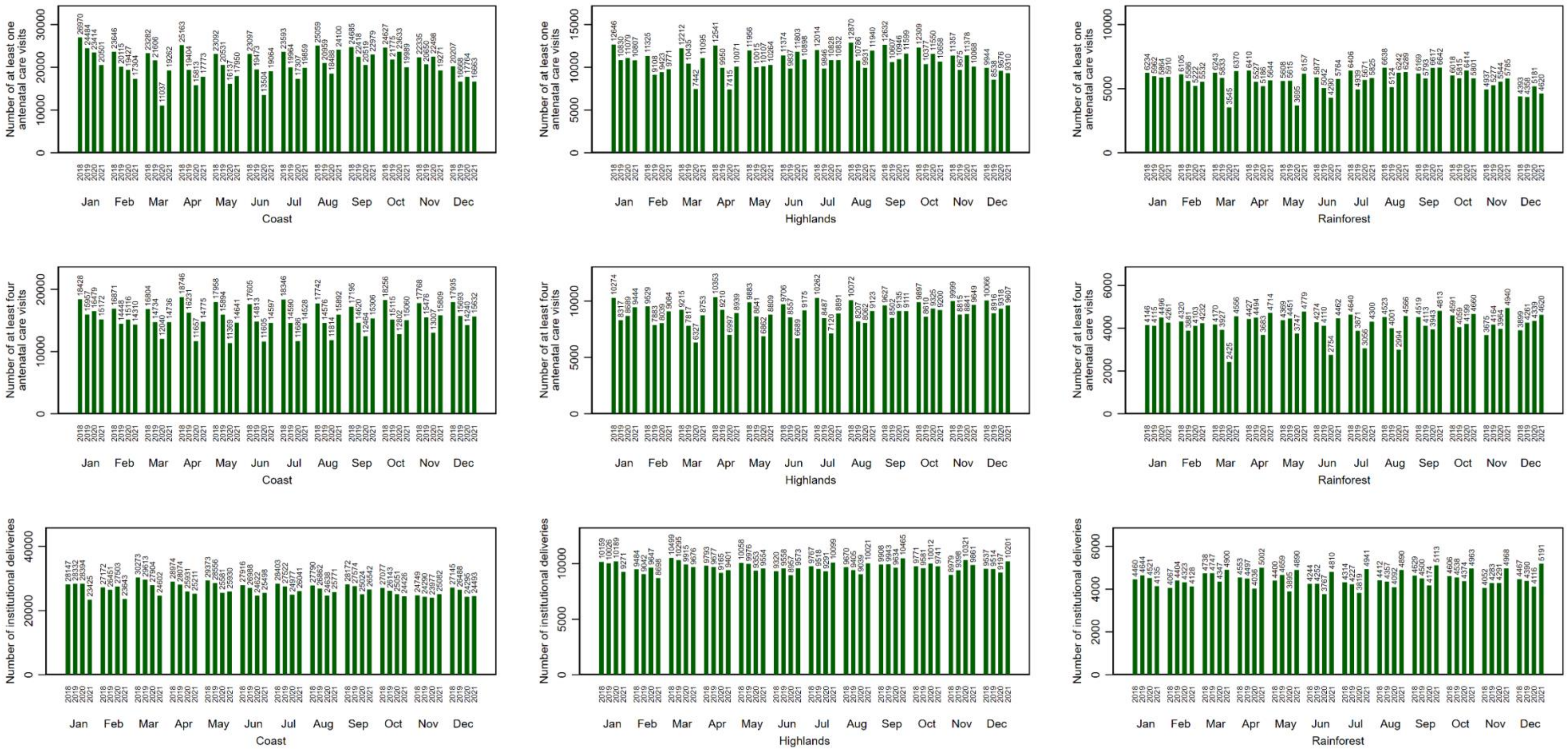

Figure S8. Monthly trend of essential maternal and child health services by natural region 2018-2021: Peru.

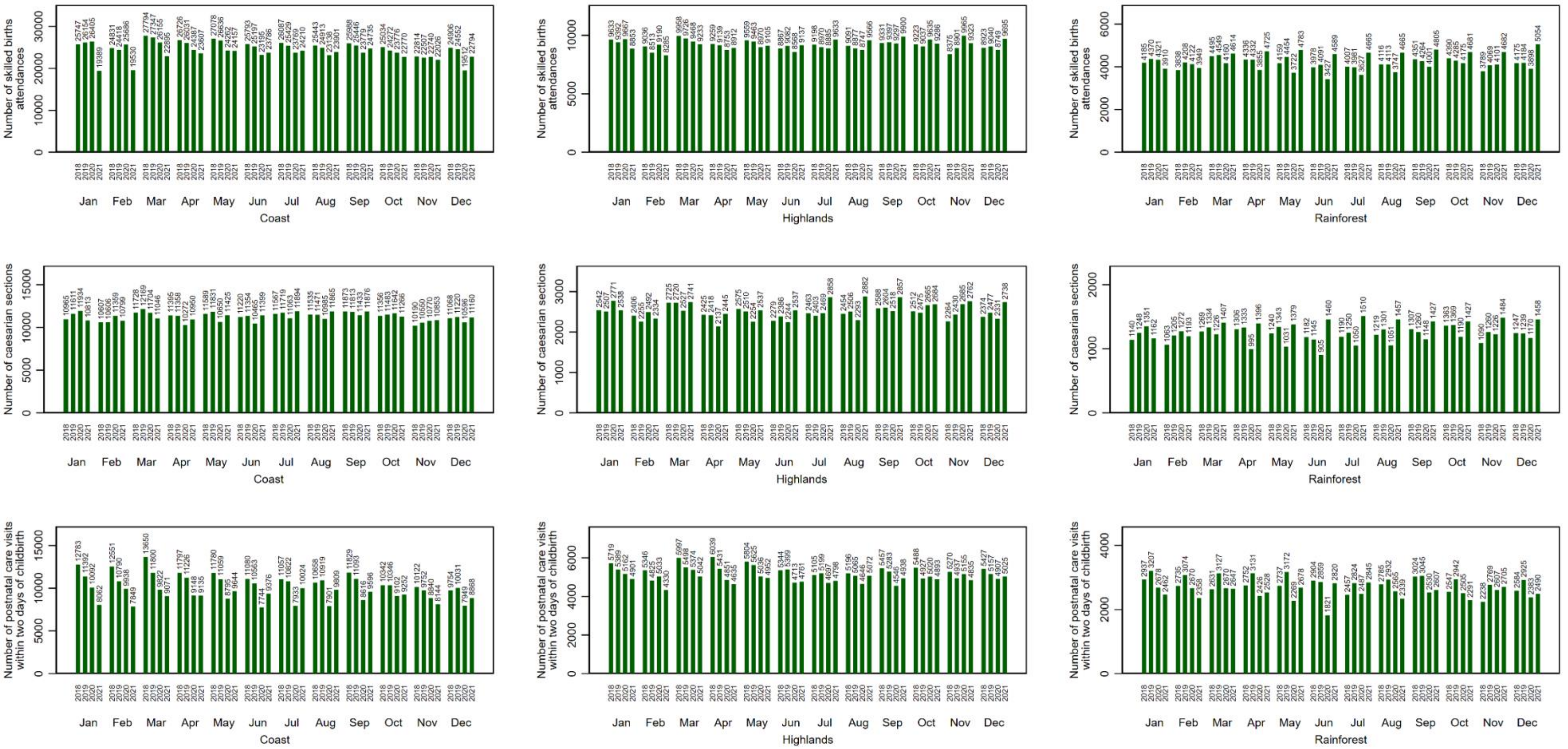

Figure S9. Monthly trend of essential maternal and child health services by natural region 2018-2021: Peru.

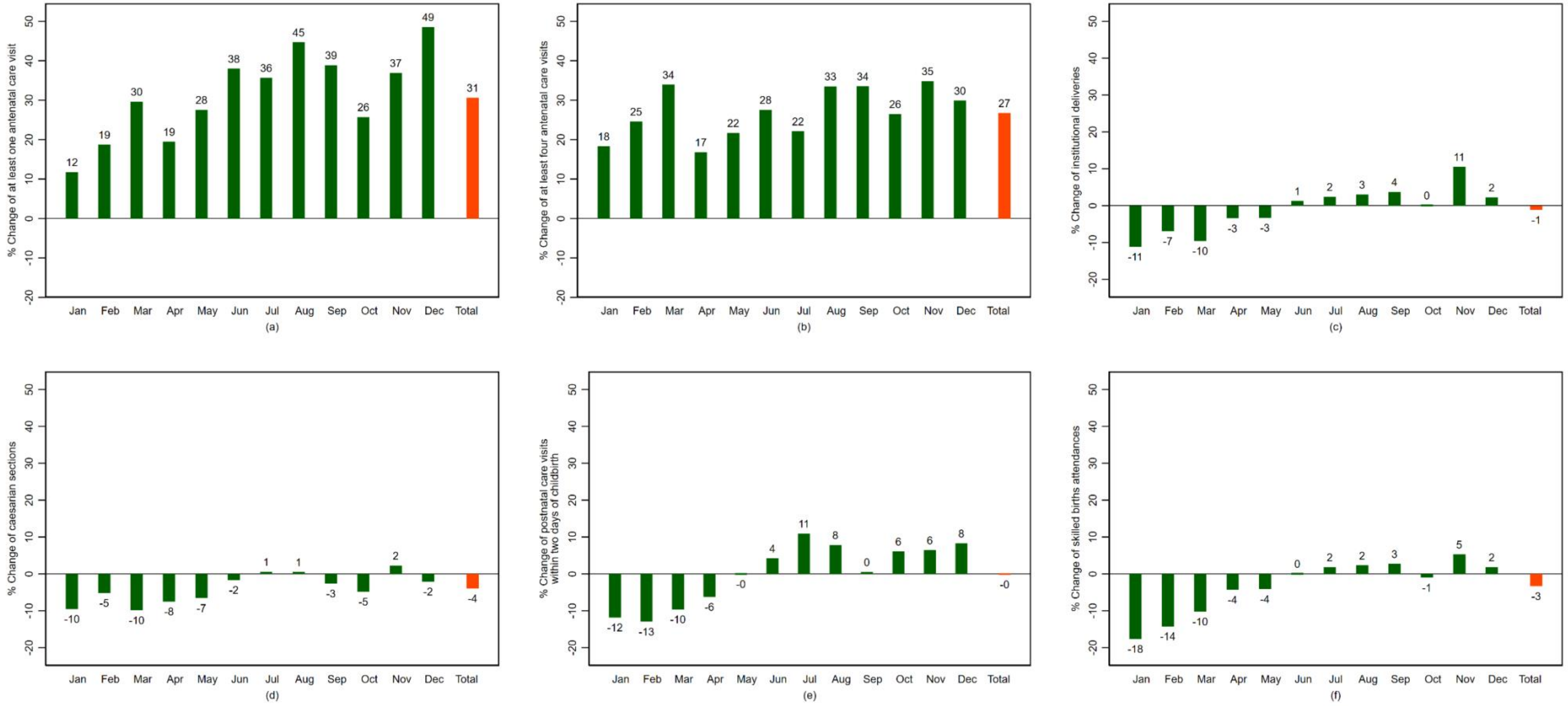

Figure S10. Percent change of essential maternal and child health services, national level, 2021. Baseline: average 2018-2019.

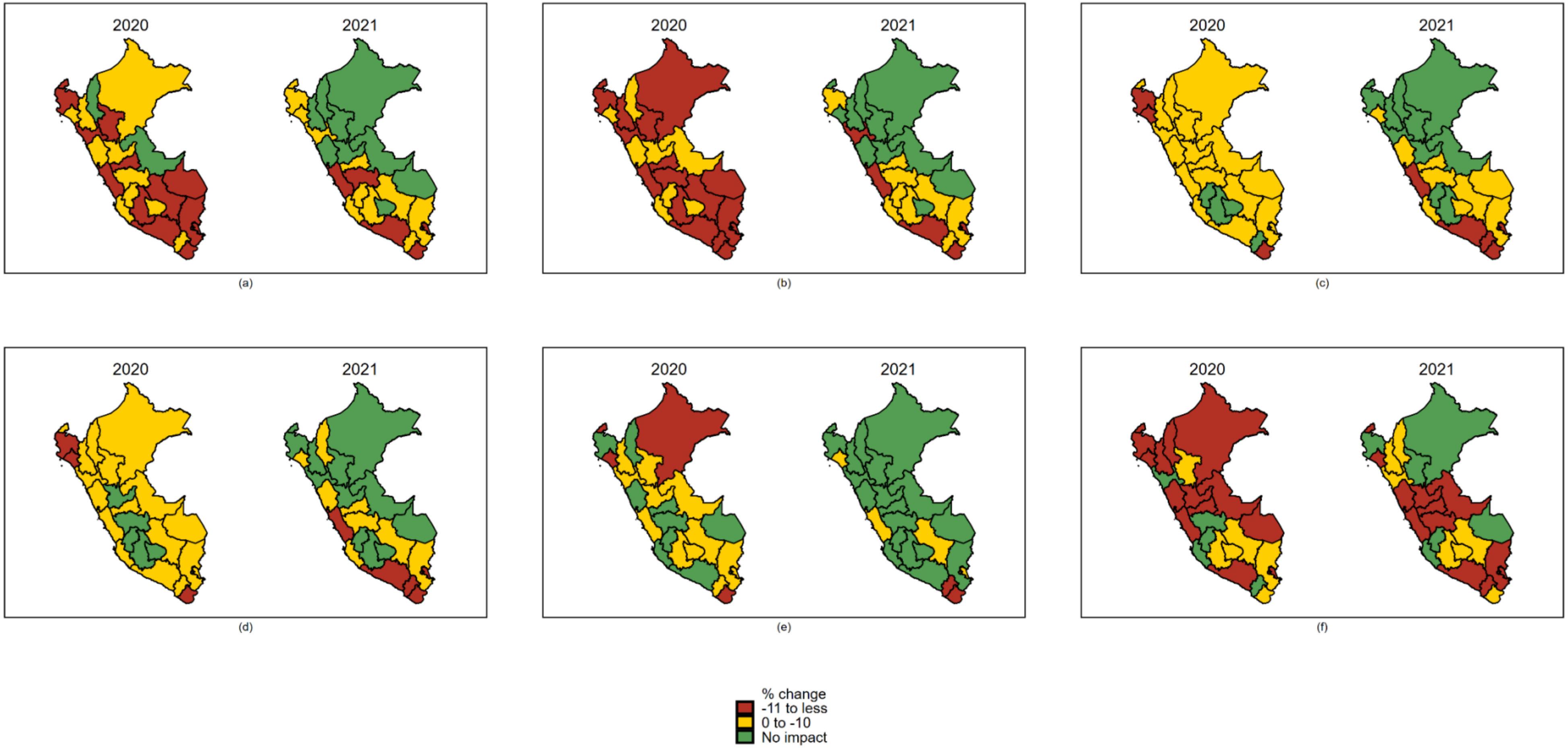

Figure S11. Percent change in the number of essential maternal and child health services at departmental level, years 2020 and 2021. Baseline: average 2018 to 2019. a. At least one antenatal care visit; b. At least four antenatal care visits; c. Institutional deliveries; d. Skilled birth attendances; e. Caesarean sections; f. Postnatal control visit within two days of childbirth.
